# The relationship between multiple chronic diseases and depressive symptoms among middle-aged and elderly populations: results of a 2009 korean community health survey of 156,747 participants

**DOI:** 10.1186/s12889-017-4798-2

**Published:** 2017-10-25

**Authors:** JooYeon Seo, BoYoul Choi, Shinah Kim, HyeYoung Lee, DongHoon Oh

**Affiliations:** 1 Institute for Health and Society, College of Medicine, Hanyang University, 222 Wangsimni–ro, Sungdong–Gu, Seoul, 04763 South Korea; 2 Department of Preventive Medicine, College of Medicine, Hanyang University, Seoul, South Korea; 3 Center for Farmers’ Safety & Health, Dankook University Hospital, Cheonan, South Korea; 4 Seulha Mental Health Clinic, Jeju, South Korea

**Keywords:** Community surveys, Comorbidity, Depressive symptoms, Epidemiologic studies, Republic of Korea

## Abstract

**Background:**

The purpose of this study was to investigate the relationship between multiple chronic diseases and depressive symptoms in middle-aged and elderly populations.

**Methods:**

This study was performed using the 2009 Korean Community Health Survey, which targeted adults over the age of 40 (*N* = 156,747 participants, 88,749 aged 40–59 years and 67,998 aged ≥60 years). The Korean version of the Center for Epidemiologic Studies Depression Scale (CES-D-K) was used as the measurement tool for depressive symptoms (CES-D-K score over 16). Multiple chronic diseases were defined as the concurrent presence of two or more chronic diseases.

**Results:**

The prevalence and risk ratios (RRs) of experiencing depressive symptoms increased in the presence of multiple chronic diseases and with the number of comorbidities. The RRs of experiencing depressive symptoms according to the presence of multiple chronic diseases were higher in the middle-aged population (adjusted RR, 1.939, 95% confidence limits (CL), 1.82-2.06) than in the elderly population (adjusted RR, 1.620, 95% CL, 1.55-1.69). In particular, middle-aged women who suffer from 4 or more chronic diseases have the highest RR (adjusted RR, 4.985, 95% CL, 4.13-6.03) for depressive symptoms.

**Conclusions:**

Multiple chronic diseases are closely associated with depressive symptoms in middle-aged and elderly populations. Given the mutual relationship between multiple chronic diseases and depressive symptoms, attention to and the assessment of depressive symptoms are needed in people with multiple chronic diseases.

**Electronic supplementary material:**

The online version of this article (10.1186/s12889-017-4798-2) contains supplementary material, which is available to authorized users.

## Background

To date, many researchers and clinicians have studied the relationship between the presence of chronic diseases and depression [1–4]. Most middle-aged or elderly patients with major chronic diseases have a higher chance of developing depression. For example, 18-28% of adults with diabetes, 16.7% of adults with hypertension experience depression. These numbers are higher than those in groups without chronic diseases [5, 6]. A previous study suggested that patients with diabetes, vascular diseases, and cancer who experience depression tend to die 5 to 10 years earlier than patients without any psychiatric diseases [7]. However, previous studies mainly studied the relationship between depressive symptoms and chronic diseases in adults over 55 years old [8–11]. Consequently, we need to figure out the mutual influence by analyzing the relationship between chronic illnesses and depressive symptoms, among others, by means of indicating deterioration, increasing disability and economic burden [2, 9, 12].

Multiple chronic diseases (MCDs) also known as comorbidity are defined as two or more chronic diseases. The prevalence of MCDs and the management of these conditions has become an important public health issue [13–16]. Up to date, several studies have investigated the impact of MCDs on depression, as well as the relationship between specific chronic diseases and depression [3, 15, 17, 18]. However, few studies have targeted middle-aged or younger populations compared with elderly populations [1, 10, 19, 20]. A previous study reported the relationship between chronic physical conditions and depression in patients aged 22–64 years and considered the differences in the age groups [19]. The study focused on each chronic disease individually and on only younger adults [19]. In addition, previous studies investigated the relationship between MCDs and depression itself, but the effect of the numbers of chronic diseases on depression has not been studied [1, 6, 21]. Hence, the effect of MCDs and the number of chronic diseases on depression in a younger population, such as a middle-aged population, has not been explicitly studied.

Therefore, we estimated the prevalence depressive of depressive symptoms and MCD, and studied the relationship between MCDs and depressive symptoms in middle-aged and elderly populations using data from a large, representative national sample obtained through a community health survey in Republic of Korea (below, Korea). The specific objectives of this study were to estimate the prevalence of depressive symptoms and MCDs and to investigate the relationship between MCDs and depressive symptoms in these age groups.

## Methods

### Data and participants

**Fig. 1:**
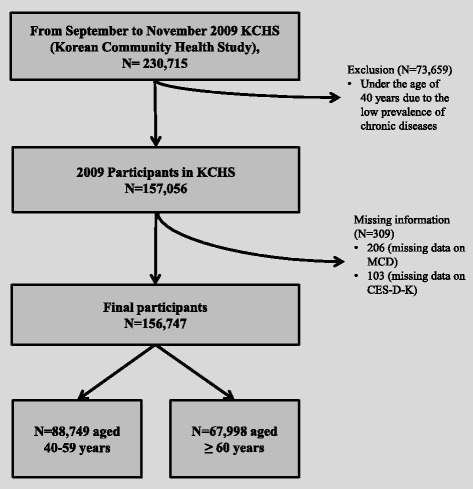
Study participants

This study was performed using the data from the Korean Community Health Survey (KCHS) conducted by the Korean Centers for Disease Control and Prevention (KCDC) in 2009. The KCHS has been a national health survey with direct face-to-face interviewing by a trained interviewer, and 253 national public health centers have participated annually since 2008. The KCHS has public confidence and represents the entire population of Korea. It is secondary data produced by the Korean government for the purpose of public use and is designed as a cross sectional study [https://chs.cdc.go.kr/chs/intro.html]. The aims of the KCHS are to calculate the health statistics of city and county units to establish regional health plans, to produce basic data to systematically assess the local health service, to expand the community surveillance infrastructure by establishing public cooperation, and to perform a standardized survey to compare the health of the population [22]. The KCHS 2009 was conducted from September to November 2009 on study participants aged 19 or older in each area who were selected by the probability proportional sampling method and the systematic sampling method. The KCHS collects detailed information, including sociodemographic characteristics, 15 types of chronic diseases in the individual’s past medical history data (defined as “diseases diagnosed by medical doctors”), lifestyle, behaviors, and health-related problems. The KCHS protocol is annually reviewed and approved by the institutional review board of the KCDC. Written informed consent was obtained from all participants in the KCHS. No additional institutional review board approval was required for this study because the data were intended for public purposes [23]. The 2009 KCHS included 230,715 participants [24]. We excluded 73,659 participants under the age of 40 years due to the low prevalence of chronic diseases in this population. In fact, as shown in previous studies, the prevalence of MCDs and chronic diseases, such as hypertension and diabetes, in individuals less than 40 years of age is relatively low [25, 26]. The prevalence of MCDs is only 0.4% in individuals in their 20s and 1.4% in those in their 30s. In contrast, the prevalence of MCDs is 11.9% in those aged 40–59 years and 46.3% in those aged ≥60 years in the 2009 KCHS. In addition, this study focused on comparing middle-aged and elderly populations. For these reasons, participants under the age of 40 years were excluded from the study. Moreover, 309 individuals who did not provide complete information on the questionnaire were excluded from this study (206 individuals had missing data on MCDs, and 103 individuals had a missing Center for Epidemiologic Studies Depression Scale (CES-D-K) score). Finally, a total of 156,747 participants (88,749 aged 40–59 years and 67,998 aged ≥60 years; 71,695 men and 85,052 women) were included in the study. The participant selection process is shown in Fig. 1.

### Sociodemographic variables

The participants were divided into age groups, namely, 40–59 years and 60 years or over, for comparisons between the middle-aged and elderly populations. Marital status was classified as follows: married, separated, divorced, widowed, and never married. Employment was classified as yes/no. Occupational categories were classified into manual, non-manual, and unemployed/others. The category of other occupations included students and housewives. Educational status was classified as not educated, elementary school, middle school, high school, and college or more. The average monthly household family income of the participants was classified as 1 million won (Korean currency unit) or less, 2 million won or less, 3 million won or less, 4 million won or less, and 4.01 million won or more. It is the monthly average of the past one year of actual income of all household members except taxes. One thousand won roughly equals one US dollar. The residence locations were classified into four groups according to the Korean administrative districts, which are based on population size: (1) county (population less than 50,000), (2) small city (population more than 50,000), (3) medium city (population more than 500,000), and (4) metropolitan area (population more than 1,000,000). All questionnaires were conducted through one-on-one interviews.

### Independent variables: Chronic diseases

The 2009 KCHS covered 15 chronic medical diseases: (1) diabetes, (2) dyslipidemia, (3) hypertension, (4) angina, (5) myocardial infarction, (6) stroke, (7) osteoporosis, (8) arthritis, (9) tuberculosis, (10) asthma, (11) hepatitis B, (12) atopic dermatitis, (13) allergic rhinitis, (14) cataract, and (15) depression. All chronic diseases were investigated with the following question: “Have you ever been diagnosed with any specific disease by a physician in your lifetime?” We used the number of comorbid chronic diseases to define the MCDs. MCDs were defined as the concurrent presence of two or more chronic diseases. Tuberculosis can be cured, but the treatment period can last for more than six months; therefore, tuberculosis affects the individual’s quality of life and is not only an endemic disease but also one of the highest public health priorities in Korea. Therefore, we included tuberculosis as an important medical disease despite it being treatable [27].

### Dependent variables: Depressive symptoms

We used depressive symptoms as a dependent variable. Commonly, depressive symptoms include low mood, feeling fatigue, anxiety, reducing self-esteem, and sleep disturbance [28]. Also, the survey about depressive symptoms is an effective tool for screening depression [29].

The 2009 KCHS used the Korean-validated 20-item version of the CES-D-K to assess depressive symptoms [30] and the definition of depressive symptoms is equal to or greater than a CES-D-K score of 16 [31]. The CES-D was not originally developed to generate a clinical diagnosis of depression, but to detect individuals with depressive symptoms. The CES-D has been translated into Korean, and its reliability and validity have been confirmed [32]. Cho et al. suggested two optimal cut-off points: one is the universal cut-off point of 16, which effectively detects and assesses ‘probable’ depressive symptoms; the other is 25, which more accurately corresponds to the DSM-III-R classification of major depression and is regarded as the cut-off point for indicating severe, ‘definite’ depression symptoms [33].

This scale is designed to identify the existence of depressive symptoms and evaluate their severity. It is a 20-item measure consisting of 4 positively worded items and 16 negatively worded items. The positive items are reverse-coded so that the scores have a potential range of 0 to 60, with higher scores indicating the presence of more depressive symptoms [23]. The internal consistency of the Korean version of the CES-D-K was verified in 1993 by Cho et al. (Cronbach’s alpha = 0.9098) [32].

### Statistical analysis

All analyses were stratified by age group for comparisons between the middle-aged and elderly populations. Chi-square tests were used to examine the significance of the differences in the sociodemographic characteristics, numbers of chronic diseases, presence of depressive symptoms and MCDs, and depression diagnosed by physicians between the age groups. The CES-D-K scores are expressed as the medians and interquartile ranges (IQRs) and compared by age group using Mann–Whitney U test. The scores were transformed to a natural logarithm because they did not follow a normal distribution.

The prevalence of depressive symptoms and the CES-D-K scores were described for each disease. The severity of the depressive symptoms according to the disease state or number of diseases was described as the median and IQR of the CES-D-K scores, and the *p*-values were calculated using Student’s t-test to compare two groups (presence of each disease or MCDs) or ANOVA (Analysis of Variance) to compare three or more groups (the groups distinguished by the number of diseases)..

We performed a multivariate logistic regression analysis to assess the relationship between depressive symptoms and each chronic disease, MCDs, and number of diseases. Multivariate logistic regression analyses were performed to calculate the adjusted risk ratios (RRs) and 95% confidence limits (CLs) by adjusting for variables affecting the depressive symptoms, such as age (years), gender (except when the results were stratified by gender), marital status, employment, occupational categorization, education, monthly income, and residence location [34], and each disease to correct for the influence of other diseases and analyze the depressive symptoms in each disease. *P* values <0.05 were considered statistically significant, and all statistical analyses were performed with SAS 9.4 (SAS Inc., Cary, NC, USA).

## Results

Table 1 shows the sociodemographic characteristics of the study population according to age group. There were statistically significant differences between the two groups in all variables (*p < 0.001*). A total of 11.88% of middle-aged individuals had MCDs, and 46.27% of the elderly population had MCDs. This value was significantly higher in elderly than that for the middle-aged population (*p < 0.001*). Approximately 1% of the middle-aged population and 9.5% of the elderly population had 4 or more chronic diseases (*p < 0.001*)*.* The prevalence of depressive symptoms in the elderly population (17.32%) was significantly higher than that in the middle-aged population (9.59%) (*p < 0.001*). However, the prevalence of depression diagnosed by physicians was 2.77% and 3.58%, respectively.

**Table 1:** Sociodemographic and clinical characteristics of the study population

	40-59 yr		≥60 yr		*p* ^*a*^
	(No. participants = 88,749)		(No. participants = 67,998)		
	*n*	%	*n*	%	
Gender					
Men	42,961	48.41	28,734	42.26	<0.001
Women	45,788	51.59	39,264	57.74	
Marital status					
With spouse	72,945	82.19	43,552	64.05	<0.001
Separated	4459	5.02	1746	2.57	
Divorced	4698	5.29	1081	1.59	
Widowed	3474	3.91	21,286	31.3	
Never married	3173	3.58	333	0.49	
Employment					
No	4527	5.1	22,956	33.76	<0.001
Yes	84,170	94.84	45,029	66.22	
Unknown	52	0.06	13	0.02	
Occupational categories					
Manual	31,572	35.57	21,798	32.06	<0.001
Non-manual	33,635	37.9	5012	7.37	
Unemployed/other^*b*^	23,490	26.47	41,175	60.55	
Unknown	52	0.06	13	0.02	
Education					
College or more	19,617	22.1	3384	4.98	<0.001
High school	36,308	40.91	8389	12.34	
Middle school	16,603	18.71	8492	12.49	
Elementary School	13,192	14.86	23,112	33.99	
Non-educated	3029	3.41	24,621	36.21	
Income (Korean 10,000won/month)^*c*^					
4.01 million won or more	17,966	20.24	4418	6.5	<0.001
4 million won or less	11,184	12.6	3097	4.55	
3 million won or less	20,672	23.29	7242	10.65	
2 million won or less	21,260	23.96	13,571	19.96	
1 million won or less	14,562	16.41	37,459	55.09	
Unknown	3105	3.5	2211	3.25	
Residence location^*d*^					
County	27,708	31.22	32,409	47.66	<0.001
Small city	26,235	29.56	17,741	26.09	
Middle city	9543	10.75	4137	6.08	
Metropolitan	25,263	28.47	13,711	20.16	
No. of chronic diseases					
0	57,127	64.37	16,685	24.54	<0.001
1	21,076	23.75	19,853	29.2	
2	7351	8.28	15,520	22.82	
3	2332	2.63	9505	13.98	
4 or more	863	0.97	6435	9.46	
Multiple chronic diseases					
No (chronic diseases 0–1)	78,203	88.12	36,538	53.73	<0.001
Yes (chronic diseases ≥2)	10,546	11.88	31,460	46.27	
Depressive symptoms					
No (CES-D-K^e^ 0–15)	80,234	90.41	56,219	82.68	<0.001
Yes (CES-D-K^e^ ≥ 16)	8515	9.59	11,779	17.32	
CES-D-K (median, IQR)	3	1-3	5	2-12	<0.001
Depression diagnosed by physicians					
No	86,291	97.23	65,567	96.42	<0.001
Yes	2458	2.77	2431	3.58	

^a^P-values were obtained using the chi-square or Mann Whitney U test with the CES-D-K scores

^b^Including housewives

^c^One thousand won roughly equals one US dollar

^d^County; population less than 50,000

Small city; population more than 50,000

Middle city; population more than 500,000

Metropolitan; population more than 1,000,000

^e^CES-D-K; Korean version of the Center for Epidemiological Studies Depression

Table 2 presents the prevalence, RRs of having depressive symptoms, and CES-D-K scores (median, IQR) for each chronic disease category. The adjusted RRs were statistically significant except for hypertension in the middle-aged population dyslipidemia, and hypertension in the elderly population. Especially, the RRs of the individuals with hypertension experiencing depressive symptoms were not significant in either group. The adjusted RRs for experiencing depressive symptoms with stroke were higher than those for any other disease in the 40- to 59-year-old population (adjusted RR, 2.336, 95% CL, 1.98-2.75) and over 60 years old (adjusted RR, 2.408, 95% CL, 2.23-2.60). Accordingly, the CES-D-K scores were the highest in the individuals with stroke (median: 8, IQR: 3–18 in the middle-aged population and median: 10, IQR: 4–20 in the elderly population). In addition, the RRs of the individuals with dyslipidemia experiencing depressive symptoms were not significant in the elderly population. Overall, the RRs of the patients with all diseases experiencing depressive symptoms were higher in the middle-aged population than in the elderly population, except for stroke and allergic rhinitis, in which the CES-D-K scores in patients with these diseases were significantly higher than in those without these diseases.

**Table 2:** Prevalence and Risk ratios of depressive symptoms according to individual chronic disease

		40-59 yr						≥60 yr					
		(No. participants = 88,749)						(No. participants = 67,998)					
		Prevalence (%)	Adjusted	95% CL^*b*^	CES-D-K^*c*^			Prevalence (%)	Adjusted	95% CL^*b*^	CES-D-K^*c*^		
			risk ratio^a^		Median	IQR	*p* ^*d*^		risk ratio^a^		Median	IQR	*p* ^*d*^
Diabetes	Yes	15.33	1.362	1.25-1.49	4	1-10	<0.001	21.18	1.21	1.14-1.28	6	2-14	<0.001
	No	9.21			3	0-8		16.63			5	2-11	
Dyslipidemia	Yes	13.4	1.274	1.18-1.38	4	1-9	<0.001	18.91	1.026	0.96-1.11	6	2-13	<0.001
	No	9.26			3	0-8		17.16			5	2-12	
Hypertension	Yes	12.1	1.066	0.99-1.14	3	1-9	<0.001	19.26	0.956	0.92-1.00	6	2-13	<0.001
	No	9.12			3	0-8		15.83			5	1-11	
Angina	Yes	22.41	1.725	1.43-2.08	5	1-14	<0.001	26.13	1.386	1.25-1.53	7	3-16	<0.001
	No	9.47			3	1-8		16.99			5	2-12	
Myocardial infarction	Yes	23.21	1.695	1.35-2.12	6	2-14	<0.001	27.53	1.555	1.39-1.74	7	2-17	<0.001
	No	9.5			3	1-8		17.02			5	2-12	
Stroke	Yes	31.21	2.336	1.98-2.75	8	3-18	<0.001	34.59	2.408	2.23-2.60	10	4-20	<0.001
	No	9.38			3	1-8		16.28			5	2-11	
Osteoporosis	Yes	20.91	1.599	1.44-1.78	6	2-14	<0.001	26.47	1.328	1.26-1.40	8	3-16	<0.001
	No	9.22			3	0-8		15.31			5	1-11	
Arthritis	Yes	18.89	1.605	1.49-1.73	5	2-12	<0.001	24.47	1.382	1.32-1.45	7	3-15	<0.001
	No	8.77			3	0-7		14.18			4	1-10	
Tuberculosis	Yes	15.87	1.542	1.36-1.75	4	1-11	<0.001	20.89	1.285	1.15-1.43	6	2-14	<0.001
	No	9.44			3	1-8		17.19			5	2-12	
Asthma	Yes	20.65	1.638	1.41-1.91	5	1-13	<0.001	27.27	1.417	1.30-1.54	8	3-17	<0.001
	No	9.44			3	1-8		16.82			5	2-12	
Hepatitis B	Yes	13.36	1.434	1.25-1.64	4	1-10	<0.001	18.99	1.297	1.10-1.53	5	2-13	<0.001
	No	9.5			3	1-8		17.3			5	2-12	
Atopic dermatitis	Yes	18.82	1.801	1.54-2.10	5	1-12	<0.001	26.83	1.525	1.31-1.78	8	2-16	<0.001
	No	9.46			3	1-8		17.18			5	2-12	
Allergic rhinitis	Yes	12.62	1.348	1.25-1.46	4	1-9	<0.001	22.72	1.378	1.24-1.53	6	2-14	<0.001
	No	9.33			3	0-8		17.11			5	2-12	
Cataract	Yes	16.2	1.28	1.11-1.47	4	1-11	<0.001	23.33	1.103	1.05-1.16	7	3-15	<0.001
	No	9.46			3	1-8		15.74			5	1-11	

^a^Multivariate logistic regression model adjusted for age (continuous variable), gender, marital status, employment, occupational categories, education, income, residence location and each disease

^b^95% CL; 95% confidence limits

^c^CES-D-K; Korean version of the Center for Epidemiological Studies Depression

^d^P-values were obtained using Student’s t-test with the CES-D-K scores transformed by natural logarithm

Table 3 shows that the prevalence, RRs for experiencing depressive symptoms, and CES-D-K scores increased in the presence of MCDs and with the number of chronic diseases (0, 1, 2, 3 and more than 4 diseases). The RRs of experiencing depressive symptoms in individuals with MCDs (yes/no) were higher in the middle-aged population (adjusted RR, 1.939, 95% CL, 1.82-2.06) than in the elderly population (adjusted RR, 1.620, 95% CL, 1.55-1.49). In particular, the RRs of experiencing depressive symptoms increased dramatically in the middle-aged patients with more than four diseases (adjusted RR, 4.465, 95% CL, 3.81-5.23). The extent of the increase in the CES-D-K score was higher in the middle-aged population than in the elderly population. In addition, we performed additional analyses stratified by gender (men and women) (Additional file 1: Table S1 and Additional file 2: Figure S1).

**Table 3:** Prevalence and Risk ratios of depressive symptoms according to the presence of multiple chronic diseases

		40-59 yr						≥60 yr					
		(No. participants = 88,749)						(No. participants = 67,998)					
		Prevalence (%)	Adjusted	95% CL^*b*^	CES-D-K^*c*^			Prevalence (%)	Adjusted	95% CL^*b*^	CES-D-K^*c*^		
			risk ratio^a^		Median	IQR	*p* ^*d*^		risk ratio^a^		Median	IQR	*p* ^*d*^
No. of diseases	0	7.67			3	0-7	<0.001	10.78			4	1-9	<0.001
	1	10.7	1.429	1.35-1.51	3	1-8		14.22	1.222	1.15-1.30	4	1-10	
	2	14.95	1.879	1.74-2.03	4	1-11		18.03	1.468	1.37-1.57	6	2-12	
	3	20.88	2.602	2.32-2.92	5	2-13		24.12	1.93	1.80-2.07	7	3-15	
	4 or more	33.72	4.465	3.81-5.23	8	3-19		32.09	2.643	2.45-2.85	10	4-19	
Multiple chronic diseases	No	8.49			3	0-7	<0.001	12.65			4	1-9	<0.001
	Yes	17.8	1.939	1.82-2.06	5	1-12		22.75	1.620	1.55-1.69	7	2-14	

^a^Multivariate logistic regression model adjusted for age (continuous variable), gender, marital status, employment, occupational categories, education, income and residence location

^b^95% CL; 95% confidence limits

^c^CES-D-K; Korean version of Center for epidemiological studies depression

^d^P-values were obtained by Student’s t-test or ANOVA using CES-D-K score transformed by natural logarithm

We investigated the differences between the three groups; 0 vs. 1 vs. 2 or more diseases (MCDs) for examining the effects of 1 single disease vs. 0. In all four groups, the RRs of having depressive symptoms and the CES-D-K scores in the single disease participants were significantly greater than in those with no chronic diseases. The RRs in the middle-aged groups were elevated compared with those in the elderly group (Additional file 1: Table S1). We illustrated that the RRs of the participants with depressive symptoms increased as the number of comorbidities increased (Additional file 2: Figure S1). As shown, as the number of comorbidities increases, the RRs increased to a greater extent in the middle-aged group than in the elderly group. The patterns between the men and women in the same age groups were similar, but the RRs of having depressive symptoms in those with 4 or more diseases was the highest in the middle-aged women (adjusted RR, 4.985, 95% CL, 4.13-6.03).

## Discussion

Our study investigated the relationship between MCDs and depressive symptoms in the largest sample of middle-aged and elderly South Koreans examined to date.

We found that MCDs were associated with increases in the prevalence, severity, and RRs for experiencing depressive symptoms in both middle-aged and elderly people. However, as the numbers of comorbidities increased, the RRs of experiencing depressive symptoms increased to a greater extent in the middle-aged population than in the elderly population. When analyzing the results stratified by gender, the patterns in the same age groups were similar between the men and women. As the number of diseases increased, as depressive symptoms increased in both men and women.

Previous studies have reported an association between higher levels of depression and the presence of MCDs [10, 11, 15, 17, 19]. For example, the results from a US study conducted through telephone interviews indicated that multiple chronic conditions, particularly pulmonary diseases, increased the risk of depression [15]. In our study, there was a high association between depressive symptoms and certain chronic diseases, particularly angina, myocardial infarction, and stroke. In particular, our study showed that the greatest RRs of experiencing depressive symptoms were observed in both middle-aged and elderly individuals with stroke.

Our results are in line with those of previous studies [8, 21, 35]. Diseases such as stroke and myocardial infarction are associated with severe and persistent symptoms, disabilities, and high mortality rates, regardless of gender and age. Numerous studies have analyzed disability and depression following stroke and myocardial infarction [36–39]. Stroke leads to post-stroke depression (PSD) following the associated physical and cognitive impairments [38, 39]. PSD is caused by various biological mechanisms, such as anatomical damage, particularly a left frontal lesion of the brain and neuronal cell damage induced by pro-inflammatory cytokines, such as interleukin (IL)-1, IL-6, and IL-8 [38]. However, the RRs of experiencing depressive symptoms did not increase in the individuals with hypertension or dyslipidemia, the latter of which is generally asymptomatic or can be prevented and controlled by medication. Taken together, our results suggest that we should consider the underlying biological mechanisms, the physical and mental impairments and the symptoms of chronic diseases when assessing depressive symptoms in patients with MCDs. Biopsychosocial health care is necessary for adults with certain chronic diseases or MCDs [16].

It is important to note that the RRs of experiencing depressive symptoms were higher in the middle-aged population than in the elderly population for all chronic diseases except stroke and allergic rhinitis. This result indicates that the difference in depressive symptoms between middle-aged individuals with and without chronic diseases is greater than that between elderly individuals with and without chronic diseases. In particular, the RRs of a middle-aged patient with 3 or more diseases experiencing depressive symptoms increased dramatically.

In fact, according to several studies, older adults with chronic diseases are less likely to report worse health-related outcomes than younger adults, including physical impairments and mental health problems, such as sleep disturbances and life stress. Younger adults had more negative perceptions and emotions related to their diseases. Moreover, an awareness of chronic diseases could also affect emotional disorders [40]. In particular, middle-aged adults have many roles in social and familial activities; therefore, disease awareness at an early age of onset, role limitations, and impairments may cause a number of psychological problems [41–43]. The depressive symptoms associated with chronic diseases have various causes, such as the increased risk of complications [4], increased economic loss due to healthcare utilization and cost [44], increased functional disabilities [45], limitations of physical activity [8], and loss of productive work time [46]. These psychosocial burdens are more prevalent in middle-aged adults than in elderly adults.

In addition, the relationship between depressive symptoms and chronic diseases is strengthened in socially active groups. This observation indicates that depression is also strongly associated with limitations in social and physical activities, as well as the psychosocial burden. As an example, a Dutch study reported that there was no relationship between depressive symptoms and diseases after adjustment for physical limitations in stroke patients [8].

Comparing men and women, the prevalence of depressive symptoms in women is higher and the RRs were similar except, the RR in the middle-aged women with 4 or more diseases was significantly higher than those in the other groups. According to several previous studies, women in the general population have more severe depressive episodes with increased functional impairment and are more likely to develop depression than men [47]. Therefore, in middle-aged women whose social functioning is important, more chronic diseases are associated with the development of increased depressive symptoms. Further studies are needed to explore the biological factors, such as hormonal changes and menopause, associated with susceptibility to depressive symptoms in middle-aged women with MCDs.

Diseases (or symptoms) that share disease-associated cellular components (genes, proteins, metabolites, miRNAs) show comorbidity [48]. The relationship between depressive symptoms and chronic diseases is bidirectional. Many symptoms of chronic diseases cause depression, but depression is also a risk factor or aggravating factor that affects a patient’s health behavior, the patient-physician relationship, self-care (e.g., diet, exercise, and smoking cessation) [2], and the biological inflammation mechanism. Therefore, new chronic diseases are likely to affect patients with depression due to poor self-management as well as the presence of biological inflammatory mediators, such as IL-6 and C-reactive protein (CRP) [38, 49]. Additionally, someone with one chronic disease has a higher chance of developing other chronic conditions because the functional and biological mechanisms are common [26, 49, 50]. Therefore, there is an increased possibility that MCDs may develop in a depressed patient [51]. Clouse et al. [35] reported that the recognition and treatment of depression in diabetic women reduced the occurrence of diabetic complications.

This study has several limitations. First, the study has a cross-sectional design. Therefore, the causal relationship between depressive symptoms and MCDs is unclear. Second, this study was a questionnaire survey conducted by a trained interviewer; therefore, only depressive symptoms were evaluated. However, the relationship between major depression and MCD could be better elucidated in future health surveys using structural diagnostic interviews conducted by psychologists or psychiatrists. Third, chronic diseases diagnosed by medical doctors were investigated; however, information bias could occur because the past medical history questionnaires were self-reported. Fourth, diseases known to be risk factors for depression, such as chronic renal failure and pulmonary diseases were excluded from the 2009 KCHS [15, 42]. In particular, Pruchno et al. [15] reported that patients with MCDs that included pulmonary diseases had the highest odds of experiencing depression.

Despite these limitations, our study has several strengths. First, this study used the data from a large population-based sample that is representative of Korea. Second, this study used the face-to-face interview method, which has a high reliability and validity for the assessment of mental disorders [52]. In addition, this study has particular significance in aspects of public health as well as clinical issues. From the clinical perspective, the careful observation and assessment of depressive symptoms should be actively pursued in health care clinics in patients with chronic diseases, particularly when the number of chronic diseases increases, from vulnerable groups, such as the middle-aged population. From the public health perspective, attention should be paid to depressive symptoms, and an environment that improves the ability of individuals suffering from MCDs to participate in social and physical activities should be created at the community level.

## Conclusions

In conclusion, the association between MCDs and depressive symptoms was constant across the transition from the middle-aged to the elderly years. Therefore, special attention should be paid to the middle-aged population with MCDs as well as the elderly population. In addition, a comprehensive clinical evaluation that considers age, gender, and the number of diseases should be performed in individuals with MCDs and depressive symptoms.

## Additional files

Additional file 1: Table 1. Prevalence and Risk ratios of depressive symptoms according to diseases number, age and gender (DOCX 19 kb)

Additional file 2: Figure 2a. Rate ratios of experiencing depressive symptoms by the number of diseases in men aged 40-59 yr. **Figure 2b**. Rate ratios of experiencing depressive symptoms by the number of diseases in women aged 40-59 yr. **Figure 2c**. Rate ratios of experiencing depressive symptoms by the number of diseases in men aged ≥60 yr. **Figure 2d**. Rate ratios of experiencing depressive symptoms by the number of diseases in women aged ≥60 yr. (PDF 108 kb)

## Abbreviations

CES-D-K: Korean version of the Center for Epidemiologic Studies Depression Scale
CL: Confidence limit
CRP: C-reactive protein
IL: Interleukin
IQR: Interquartile range
KCDC: Korean Centers for Disease Control and Prevention
KCHS: Korean Community Health Survey
MCDs: Multiple chronic diseases
PSD: Post-stroke depression
RR: Risk ratio
