# Supplementary material for: The relationship between multiple chronic diseases and depressive symptoms among middle-aged and elderly populations: results of a 2009 korean community health survey of 156,747 participants
Source: BMC Public Health. 2017 Oct 25;17:844. doi: 10.1186/s12889-017-4798-2 (PMC5657127; doi:10.1186/s12889-017-4798-2)
Supplement: Supplementary file 1 — Prevalence and Risk ratios of depressive symptoms according to diseases number, age and gender (DOCX 19 kb) [file 12889_2017_4798_MOESM1_ESM.docx]

| - [**supplementary**](http://endic.naver.com/enkrEntry.nhn?entryId=4aea0ecd1e7f44d8a1f78298ead9b7e2)   Additional file 1: Table 1. Prevalence and Risk ratios of depressive symptoms according to diseases number, age and gender | | | | | | | | |  |
| --- | --- | --- | --- | --- | --- | --- | --- | --- | --- |
| Subgroups | | |  | Adjusted | 95% CL*^b^* | CES-D-K*^c^* | | |  |
| Age group | Gender | No. of chronic diseases | Prevalence (%) | risk ratio^a^ |  | Median | IQR | *p^d^* |  |
| 40-59 yr  (No. participants=88,749) | Men | 0 | 6.17 |  |  | 2 | 0-6 | <0.001 |  |
|  |  | 1 | 8.6 | 1.429 | 1.31-1.56 | 3 | 0-7 |  |  |
|  |  | 2 or more | 14.65 | 2.227 | 2.01-2.48 | 4 | 1-10 |  |  |
|  | Women | 0 | 9.14 |  |  | 3 | 1-8 | <0.001 |  |
|  |  | 1 | 12.71 | 1.422 | 1.32-1.53 | 4 | 1-10 |  |  |
|  |  | 2 or more | 20.11 | 2.221 | 2.01-2.42 | 6 | 2-13 |  |  |
| ≥60 yr  (No. participants=67,998) | Men | 0 | 8.21 |  |  | 3 | 1-7 | <0.001 |  |
|  |  | 1 | 10.49 | 1.207 | 1.09-1.34 | 3 | 1-8 |  |  |
|  |  | 2 or more | 15.79 | 1.739 | 1.58-1.91 | 4 | 1-11 |  |  |
|  | Women | 0 | 14.39 |  |  | 5 | 2-11 | <0.001 |  |
|  |  | 1 | 17.53 | 1.216 | 1.17-1.32 | 6 | 2-12 |  |  |
|  |  | 2 or more | 25.84 | 1.848 | 1.71-1.99 | 8 | 3-16 |  |  |

| *^a^* Multivariate logistic regression model adjusted for age (continuous variable), gender, marital status, employment, occupational categories, education, income and residence location. | | | | | | | | | |
| --- | --- | --- | --- | --- | --- | --- | --- | --- | --- |
| *^b^* 95% CL; 95% confidence limits |  |  |  |  |  |  |  |  |  |
| *^c^* CES-D-K; Korean version of the Center for Epidemiological Studies Depression scale. | | |  |  |  |  |  |  |  |
| *^d^* P-values were obtained using an ANOVA with the CES-D-K scores transformed by natural logarithm. | | | | |  |  |  |  |  |
