# Supplementary material for: The relationship between multiple chronic diseases and depressive symptoms among middle-aged and elderly populations: results of a 2009 korean community health survey of 156,747 participants
Source: BMC Public Health. 2017 Oct 25;17:844. doi: 10.1186/s12889-017-4798-2 (PMC5657127; doi:10.1186/s12889-017-4798-2)
Supplement: Supplementary file 2 — Rate ratios of experiencing depressive symptoms by the number of diseases in men aged 40-59 yr. Figure 2b. Rate ratios of experiencing depressive symptoms by the number of diseases in women aged 40-59 yr. Figure 2c. Rate ratios of experiencing depressive symptoms by the number of diseases in men aged ≥60 yr. Figure 2d. Rate ratios of experiencing depressive symptoms by the number of diseases in women aged ≥60 yr. (PDF 108 kb) [file 12889_2017_4798_MOESM2_ESM.pdf]

Additional file: Figure 1. Risk ratios of depressive symptoms by the number of diseases stratified by age and gender

		40-59 yr (No. participants=88,749)				≥60 yr (No. participants=67,998)			
		Men		Women		Men		Women	
		Adjusted risk ratio[a]	95% CL[b]	Adjusted risk ratio[a]	95% CL[b]	Adjusted risk ratio[a]	95% CL[b]	Adjusted risk ratio[a]	95% CL[b]
	0								
	1	1.432	1.31-1.57	1.430	1.33-1.54	1.212	1.10-1.34	1.221	1.21-1.33
No. of diseases	2	1.898	1.67-2.16	1.885	1.71-2.08	1.502	1.35-1.68	1.447	1.33-1.58
	3	2.918	2.42-3.52	2.459	2.13-2.84	1.769	1.55-2.02	1.971	1.81-2.15
	4 or more	3.57	2.66-4.80	4.985	4.13-6.03	2.857	2.45-3.33	2.617	2.39-2.87

[a] Multivariate logistic regression model adjusted for age (continuous variable), gender, marital status, employment, occupational categories, education, income and residence location.
[b] 95% CL; 95% confidence limits

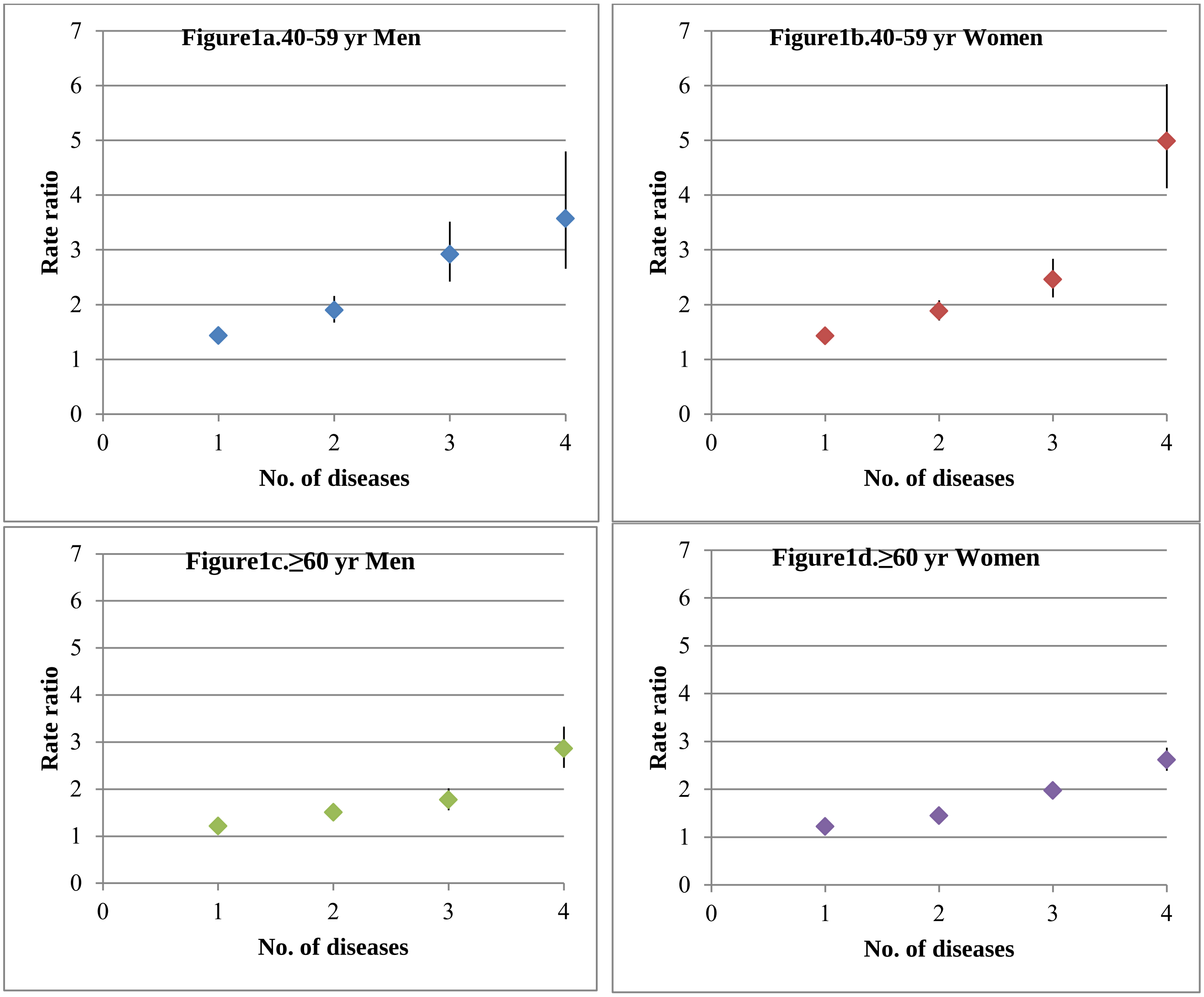

Figure legends

Supplementary figure1 a. Rate ratios of experiencing depressive symptoms by the number of diseases in men aged 40-59 yr.

Supplementary figure1 b. Rate ratios of experiencing depressive symptoms by the number of diseases in women aged 40-59 yr.

Supplementary figure1 c. Rate ratios of experiencing depressive symptoms by the number of diseases in men aged ≥60 yr.

Supplementary figure1 d. Rate ratios of experiencing depressive symptoms by the number of diseases in women aged ≥60 yr
